# Segregation of the *COL6A2* Variant (c.1817-3C>G) in a Consanguineous Saudi Family with Bethlem Myopathy

**DOI:** 10.3390/genes15111405

**Published:** 2024-10-30

**Authors:** Hitham Aldharee, Hamdan Z. Hamdan

**Affiliations:** Department of Pathology, College of Medicine, Qassim University, Buraidah 51452, Saudi Arabia; h.abualbasher@qu.edu.sa

**Keywords:** Bethlem myopathy, child, *COL6A2*, COL6-RD, muscular dystrophy, genetics, Saudi Arabia

## Abstract

**Introduction:** Bethlem myopathy is a rare genetic disease caused by a variant mapped to 21q22, which harbors the collagen type VI alpha 2 chain *(COL6A2)* and collagen type VI alpha 1 chain (*COL6A1)* genes, and 2q37, which harbors the collagen type VI alpha 3 chain (*COL6A3*) gene. Disease onset can occur at any age, and the symptoms are related to those of muscular dystrophy. Since Bethlem myopathy is a rare disease, no previous studies have been conducted in Arab countries, including Saudi Arabia. Its variable presentation of nonspecific muscular contractions and severity represents a diagnostic dilemma. **Case presentation:** Here, we report a Saudi pediatric patient, who is 9 years old (proband), brought to the pediatric clinic of King Saud’s Hospital by his mother. The boy presented with difficulty standing, walking, and running with his classmates and unaffected siblings. He has a younger sibling, aged 6 years old, who reported having a limping gait and difficulty bending his right knee. Laboratory results for the proband were unremarkable except for a slight increase in creatine kinase (CK). Whole-exome sequencing (WES) was performed for five family members, including the proband and his symptomatic brother, their mother and two asymptomatic siblings. A very rare 3′ splice site acceptor intronic variant, NM_001849.4: c.1817-3C>G, located three nucleotides before exon 25, was identified in *COL6A2*. Bioinformatics tools (SpliceAI, dbscSNV, FATHMM-MKL, and MaxEntScan) predicted this variant as pathogenic. The proband and his 6-year-old sibling presented a homozygous genotype for the variant, whereas the mother and one asymptomatic sibling were heterozygous, and the other sibling carried homozygous wild-type alleles. **Conclusions:** This is the first study to report a case of Bethlem myopathy confirmed by WES in Saudi Arabia and all Arab nations. The identified variant is rare, and its segregation pattern suggests autosomal recessive inheritance. The segregation pattern and bioinformatics tool results may qualify this variant to be annotated as pathogenic, addressing the reported uncertainty of its classification. Our findings contribute to linking and filling the knowledge gap of diagnosing and managing patients with collagen VI-related myopathies, providing greater clinical and genetic understanding to the existing knowledge.

## 1. Introduction

Bethlem myopathy (BM; MIM #158810) and Ulrich congenital muscular dystrophy (UCMD; MIM #254090) fall under the category of congenital muscular dystrophy (CMD), which defines a set of MDs that become evident at or near birth [1]. BM and UCMD are very rare inherited muscle disorders caused by variants in one of the genes responsible for collagen VI alpha chain synthesis (*COL6A1*, *COL6A2*, *COL6A3*) [2,3]. Different variants have been associated with BM, namely missense, nonsense, splice site, and Indels Table 1, constraining the normal expression and/or function of alpha chain proteins. BM has a prevalence of 0.7 in 100,000 people, while UCMD affects 0.3 in 100,000 [4]. Patients of any age can present with symptoms, including muscle weakness, joint laxity, hypotonia, and contractures in the hips and ankles [2,3]. Nevertheless, mild symptoms and slow progression are noticed in patients with BM. Since collagen VI is found in the skin, an unusual skin appearance can also be observed in BM patients.

BM is usually inherited in an autosomal dominant pattern, where a single copy from either parent is sufficient to cause the disease. However, it can also be inherited in an autosomal recessive pattern [2,3,5,6]. In some cases, the patient’s parents were asymptomatic, which could indicate that either the parents were mildly affected or the defect in the gene originated in the affected children.

Duchenne muscular dystrophy (DMD; OMIM #310200) is another rare, life-threatening neuromuscular disease that is inherited as an X-linked disease and affects the normal physiology of the muscle, similar to Bethlem myopathy. However, DMD is caused by variants in the *DMD* gene, which encodes the dystrophin protein [7,8]. Duplication and deletion are the most common alterations in the DMD gene, particularly in the hot-spot region (exon 44–55). Consequently, patients with DMD lack normal expression of the dystrophin protein, which leads to a clinical picture characterized by progressive muscle degeneration and rapid deterioration in ambulatory activities, as well as cardiac and respiratory abnormalities.

In Saudi Arabia and Arab nations, hereditary diseases are poorly investigated although they are more common than in other countries [9]. Ethnicity and social factors, namely high consanguinity rates, play a significant role in this issue [9,10,11]. The diagnosis of genetic diseases mainly depends on the patient’s clinical presentation and family history, which increases the possibility of late diagnosis or misdiagnosis. In the case of rare diseases such as BM and DMD, the signs and symptoms may overlap and lead to improper medical decisions. This study presents a rare family case of BM that was initially misdiagnosed as DMD. We report the proband’s genetic findings, characterize and compare genotype–phenotype correlation with reported cases, and discuss the family’s history of the disease.

## 2. Case Report

The proband is a 9-year-old boy who presented to the pediatric clinic of King Saud Hospital, Unaizah-Al Qassim, in August 2020 with symptoms of muscular dystrophy. The mother described the prenatal period as unremarkable, and he was delivered full-term by unassisted vaginal delivery, which was uneventful. During his neonatal period, no hypotonia was observed. The child supported his neck at around 5 months of age and was able to walk independently at around 19 months of age.

At age 4, his mother noticed that he became fatigued easily when playing with his peers and siblings and sometimes experienced repeated falls. At age 6, he experienced difficulties in standing, walking, and running. He was able to climb stairs on his own but with slight difficulty, and he would get up from the floor using his arms. During school competitions, he always lags and is slower than his peers.

On examination, the proximal muscles of the lower limbs were mildly weak, mainly the hip extensor muscle group, with a power grade of (4/5) on the Medical Research Council (MRC) scale [12], with no muscular atrophy observed. The left ankle had a slight contracture affecting the dorsiflexion action of the joint. Gower’s sign was positive [13]. The distal lower limb muscles were normal in power and shape, similar to the bilateral upper limbs and shoulder muscles. Cranial nerves and memory and cognition upon testing were intact, and no sensory loss was detected. The skin examination revealed no scars, keloid, follicular keratosis, or other clinical skin signs or abnormalities. Upon examination, there were no signs/symptoms suggestive of cardio-respiratory abnormalities. The echocardiogram and electrocardiogram (ECG) were normal. The serum creatine kinase (CK) level was abnormally high at 335 U/L (reference range 39–308 U/L).

**Figure 1:**
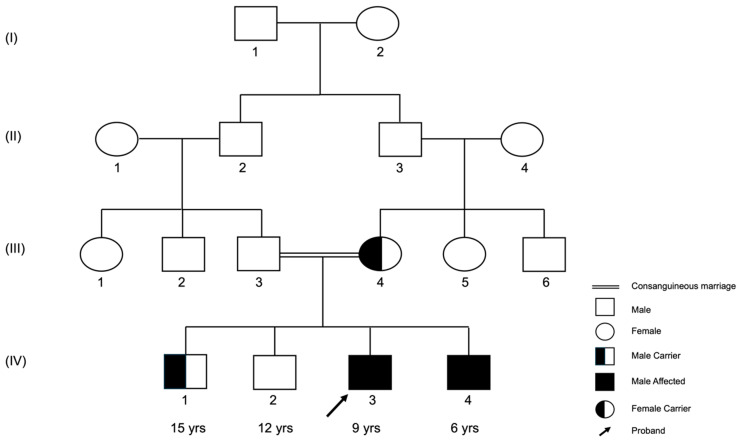
Pedigree analysis of the family carrying the variant NM_001849.4: c.1817-3C>G in *COL6A2*.

An investigation of the family’s history revealed that the parents are first-degree cousins with four male offspring. The two oldest sons were asymptomatic. However, the youngest exhibited limping gait and difficulty bending his right knee, in addition to muscle pain and fatigability similar to those reported in the proband (Figure 1). The serum CK level for the youngest sibling was 192 U/L (reference range 39–308 U/L). There is no history of similar conditions in close family relatives from either side. Informed consent for diagnostic and research purposes was obtained from the mother and the four children. Peripheral blood samples and clinical data were subsequently collected from the family members other than the father, who was not available to participate in the study.

### 2.1. Extraction of Genomic DNA and Whole-Exome Sequencing

Each participant’s genomic DNA was extracted from peripheral venous blood (2 mL), which was collected using ethylenediaminetetraacetic acid (EDTA) as an anticoagulant. Following extraction, the DNA was enriched with the MGIEasy Exome Capture V5 probe kit (MGI-Tech Co., Ltd., Shenzhen, China). The MGI-DNBSEQ Tech platform was employed to sequence the generated DNA libraries. Whole-exome sequencing (WES) services were provided by a commercial genomic laboratory accredited by the College of American Pathology (CAP). Comprehensive clinical and bioinformatics reports_which include variant genomic location, variant frequency in the Genome Aggregation Database (gnomAD), and examination of variant reports submitted to bioinformatics databases such as ClinVar and Leiden Open Variation Database (LOVD)_were provided and interpreted according to the American College of Medical Genetics and Genomics (ACMG) guidelines [14].

### 2.2. Ethical Consideration and Consent for Participation and Publication

This project received ethical approval from the Regional Research Ethics Committee, Qassim Province (Approval Number: 607/44/17554). Signed informed consent was obtained from the mother for herself and her children after informing her of the study objectives, including consent for publication.

## 3. Results

### 3.1. The (c.1817-3C>G) Variant Was Recessively Inherited in the Family

**Figure 2:**
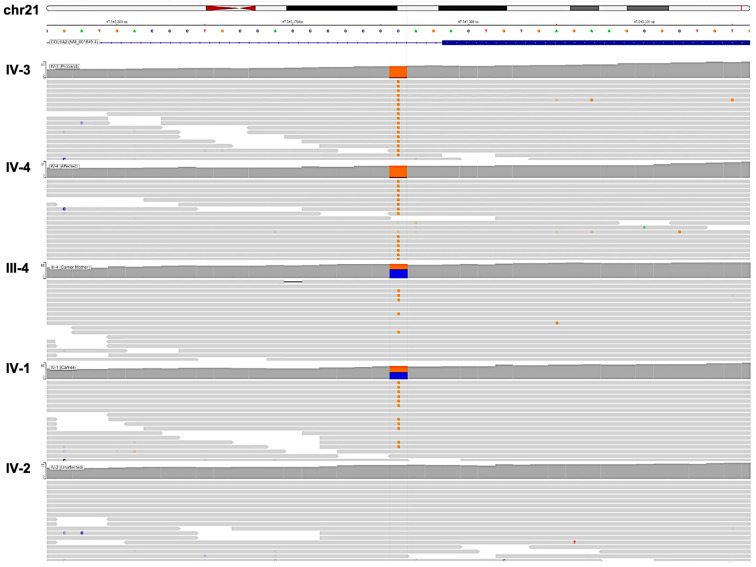
Visualization of the BAM files extracted from whole-exome sequencing (WES) confirming the (NM_001849.4: c.1817-3C>G) variant segregation in the family. The human chromosome (chr21) and the collagen type VI alpha 2 chain (*COL6A2)* gene transcript (NM_001849.4) are indicated. The human reference genome (GRCh37.P13) was used for data alignments and variant recall. The proband (IV-4) and the symptomatic sibling (IV-3) display homozygous guanosine ‘G’ alleles, shown in orange. The mother (III-4) and the asymptomatic carrier (IV-1) are heterozygous and carry both alleles. IV-2 indicates that an unaffected sibling inherited the wild-type cytosine ‘C’ alleles, shown in blue. Visualization of BAM files was performed using Integrative Genomic Viewer (IGV-Web app version 1.13.12), an open-source genome browser and visualization tool.

Upon analyzing the WES findings for all genes, particularly the targeted genes (*COL6A1*, *COL6A2*, *COL6A3*, *DMD*, *HNRPDL*, *FSHMDA1*, and *LAMA2*), the results revealed that the proband (IV-3) and his symptomatic brother (IV-4) were homozygous for the NM_001849.4: c.1817-3C>G variant in *COL6A2* (Figure 2,IV-3 and IV-4). The genomic analysis of the mother (III-4) revealed that she was an asymptomatic heterozygous carrier for this variant (Figure 2, III-4). Further analysis revealed that one sibling (IV-1) was an asymptomatic heterozygous carrier for the same variant (Figure 2, IV-1), whereas another sibling (IV-2) was negative for the variant (Figure 2, IV-2). These findings confirm the autosomal recessive inheritance of this variant. A genomic analysis for the father (III-1) was missing because the father was unavailable for genotyping in this study.

### 3.2. The (c.1817-3C>G) Variant Is Predicted to Be an Intronic and Likely Pathogenic

The genomic analysis of this variant revealed its chromosomal location to be chr21: 47,545,376. Moreover, it falls within intron 24 of the *COL6A2* gene transcript, just three nucleotides before the start of exon 25, and its SNP identifier is rs112645828 (Figure 2).

To predict the effect of the splice site variant NM_001849.4: c.1817-3C>G, we conducted bioinformatics analysis using SpliceAI, dbscSNV, FATHMM-MKL, and MaxEntScan [15,16,17,18], accessed through the varSome platform [19]. Each tool utilizes distinct algorithms and technology: Briefly, SpliceAI utilizes a 32-layer neural network based on deep learning and uniquely validates splice site variants in RNA-seq data, predicting the impact on exon gain or loss. dbscSNV predicts the single-nucleotide variant (SNV) within consensus regions of splice sites (−3 to +8 at the 5′ site and −12 to +2 at the 3′ site) using a specialized model. FATHMM-MKL employs machine learning with multiple kernel learning sets to predict SNV effects in coding and noncoding regions, using pathogenic data from the Human Gene Mutation Database and control data from the 1000 Genomes Project. MaxEntScan (MES) applies the maximum entropy principle to evaluate the impact of SNV at both the 5′ and 3′ splice sites. All tools indicated that the variant had a deleterious effect, as shown in Table 1.

**Table 1:** Prediction of the pathogenicity of the SNV (NM_001849.4: c.1817-3C>G) using the bioinformatics tools SpliceAI, dbscSNV, FATHMM-MKL, and MaxEntScan.

SNV rs112645828	SpliceAI	dbscSNV	FATHMM-MKL	MaxEntScan
Reported score	0.650	0.998	0.991	4.677
Interpretation	Acceptor loss	Pathogenic supporting	Pathogenic supporting	Pathogenic supporting

## 4. Discussion

This study described the first case of Bethlem myopathy in Saudi Arabia according to the disease progression, mild symptoms, and the slight elevation of the proband’s CK, confirmed by WES. The main finding of this study is that the homozygous (GG) splice site 3′ acceptor variant (NM_001849.4: c.1817-3C>G), rs112645828 in intron 24 of the *COL6A2* gene, was found in the two symptomatic siblings, aged 9 and 6 years. The results of the asymptomatic siblings and the mother indicated a segregation pattern of this variant in the family. The mother and one asymptomatic sibling were found to be heterozygous (CG) for this SNV. The other asymptomatic sibling was found to have the homozygous wild-type allele (CC) of this SNV. Notably, the proband and his siblings are the children of a consanguineous marriage, and the parents themselves are first-degree cousins. This finding is supported by a series of reports from a genetic testing center in Germany, CENTOGEN-AG, at the ClinVar website [20], which includes many cases of BM patients with a homozygous genotype (GG) for the same variant (NM_001849.4: c.1817-3C>G). However, Lampe et al. in the UK reported the same variant (NM_001849.4: c.1817-3C>G) in one symptomatic patient with a heterozygous genotype (CG), which indicates an autosomal dominant inheritance pattern [21]. These discrepancies in the literature may be due to interethnic variation.

At the molecular level, this SNV of *COL6A2* (NM_001849.4: c.1817-3C>G) is located at intron 24 at the third nucleotide before exon 25, and it is annotated in Ensembl as a splice 3′ acceptor variant [22]. Nucleotide substitution at the splice site is known to be associated with either intron retention or exon skipping, which can be partial or complete [23]. In either case, the sequence of the protein is altered. Hence, its folding and function will also change. Therefore, this variant, with its impact on the translated protein, is considered a loss-of-function variant [24]. In our case, exon 25, which is likely to be affected, encompasses 50 amino acids, from amino acid number 606 to amino acid 656, which participate in the formation of the second Von Willebrand factor (VWFA-2) domain. This domain contains 190 amino acids, from amino acid number 615 to amino acid number 805 [25]. Therefore, exon 25 contributes approximately 20% of the total amino acid content of VWFA-2 [26]. Notably, the COL6A2 protein contains three VWFA domains, and VWFA-2 is the largest. This VWFA-2 domain is particularly important for crosslinking with other alpha chain proteins, namely COL6A1 and COL6A3, and for fully assembling the collagen type IV protein [27]. This function may provide the rationale for why this variant is labeled as pathogenic. Moreover, some molecular genetics databases classify this variant as pathogenic, including the ClinVar database and Leiden Open Variation Database (LOVD) [20,28]. In this case, the classification is consistent with the latest updated American College of Medical Genetics and Genomics (ACMG) guidelines [14], in which this variant meets one criterion for moderate evidence of pathogenicity, which is its very rare global allele frequency of 3.992 × 10^−6^, as reported in gnomAD [29], in addition to three criteria for supporting evidence of pathogenicity: segregation of the homozygous variant in symptomatic family members only, heterozygosity in asymptomatic carriers, and the consensus of four in silico tools, namely, SpliceAI v1.3.1, dbscSNV v1.1, FATHMM-MKL v2.3, and MaxEntScan v1.1 [15,16,17,18] on the pathogenicity of this variant. Finally, the recent reporting of this variant showed that it was linked with a myopathy related to *COL6A2* [21]. Together, these criteria suggest that the current studied variant is a pathogenic variant.

Many variations have been reported to be associated with Bethlem myopathy; see Table 2. All the reported cases in *COL6A2* had missense or splice site variants. Compared to the case reported [30], our case shows similar phenotypic features of proximal lower limb muscle weakness, ankle contractures, and ambulation preservation. There were no observed cardiac or respiratory problems, consistent with all published cases of BM; see Table 2. Although c.1817-3C>G is predicted to cause the deletion of exon 25 of CLO6A2 and c.1000-2A>G was shown in [30] to affect exon 11, they both share a genotype–phenotype correlation.

The creatine kinase (CK) level is slightly elevated in our reported case. Of note is that the CK level in most reported cases of BM ranges from normal to slightly elevated. It has been reported that CK levels do not correlate with disease severity [42,43]. In contrast, in spinal muscular atrophy (SMA), a motor neuron disease, CK levels are consistently elevated and have a prognostic value [44].

To the best of our knowledge, this is the first study reporting this variant (NM_001849.4: c.1817-3C>G) of *COL6A2* as related to BM in Saudi Arabia and all Arabic countries, the inhabitants of which are primarily of Arabic ethnicity, representing approximately 5.7% (464,68 million inhabitants) of the entire world population. The rate of consanguinity in Saudi Arabia is very high; on average, it is estimated to be 56% and may exceed 80% in some local communities [45,46]. Consanguineous marriages may unmask known autosomal recessive diseases that may not have appeared in the last two generations of a family. Moreover, the unity and accumulation of heterozygous variant genotypes may also lead to the emergence of rare unknown autosomal recessive diseases in a consanguineous family [47]. The emergence of the whole-exome sequencing (WES) technique, which is based on next-generation sequencing, has aided in detecting variants associated with Mendelian inherited diseases [48]. Nevertheless, rare diseases such as Bethlem myopathy, characterized by variable severity and nonspecific muscular contractures, are difficult to diagnose and may overlap with other muscular dystrophy diseases, such as Duchene muscular dystrophy and Becker disease [49]. Therefore, we highly recommend genetic testing based on these cases, which is not a routine test in Saudi Arabia, as it will help clinicians shortlist, diagnose, and manage rare diseases.

## 5. Conclusions

In summary, we report a consanguineous Saudi family with BM caused by a rare, likely pathogenic SNV in *COL6A2* confirmed using WES. The segregation pattern is consistent with that of autosomal recessive inheritance, which is the most common type of genetic disease in the country. The genotype–phenotype correlation is consistent with previously reported cases and suggests that our case is to be identified as a BM one. Further longitudinal studies are needed, to explore and confirm the impact of this SNV on the expression of the *COL6A2* gene and the translated protein. These can be conducted by applying the RNA sequencing technique, the minigene assay and protein functional assays.

## Figures and Tables

**Table 2:** Reported cases of Bethlem myopathy (BM). This table presents various genetic variants described in the literature and their phenotypic correlations.

Case	Gene	Variant	Type	Genotype	Lost Ambulation	Walking Abnormality	Respiratory Symptoms	Muscle Weakness and Contractures
[31] (Wilpert et al., 2024)	*COL6A1*	c.117_119delCCT	In-Frame deletion	Hh	Not reported	Truncal pendulum movements during walking.	Not reported	Elbow and long finger flexor contractures. Mild proximal muscle weakness
[32] (Bardakov et al., 2021)	*COL6A1*	c.227 + 2T>C	Splice site	HH	Not reported	Not reported	Not reported	Early-onset severe proximal joint contractures and distal joint hypermobility
[33] (Kachuei et al., 2024)	*COL6A2*	c.385C>T	Missense	HH	yes	Waddling gait and	Not reported	Proximal lower limb weakness, a positive Gowers’ sign, lumbar hyperlordosis and absent myotatic reflexes
[33] (Baker et al., 2007)	*COL6A2*	c.1000-2A>G	Splice site	Hh	Not reported	Not reported	Not reported	Proximal lower limb muscle weakness and contractures of elbow, knee, and ankles
[6] (Elmas and Gogus, 2018)	*COL6A2*	c.2584C>T (p.Arg862Trp)	Missense	Hh	Not reported	Not reported	Not reported	Joint contractures, finger flexors, neuromotor developmental delay
[34] (Kutluk et al., 2021)	*COL6A2*	c.2096G>A	Missense	Hh	Not reported	Not reported	Not reported	Predominant proximal muscle weakness, contractures at metacarpals, distal hyperextensibility of fingers
[34] (Kutluk et al., 2021)	*COL6A2*	c.2096G>A	Missense	Hh	Not reported	Not reported	Not reported	Predominant proximal muscle weakness, distal hyperextensibility of fingers
[35] (Oros et al., 2023)	*COL6A2*	c.784G>T	Missense	Hh	Not reported	Difficulty to walk independently and running	Not reported	Progressive motor deficit involving all four limbs
[2] (Gualandi et al., 2009)	*COL6A2*	Q819X; R366X	Missense	HH	Not reported	Not reported	Not reported	Proximal lower limb muscle weakness and contractures of fingers flexors muscles
[36] (Stavusis et al., 2020)	*COL6A3*	c.7447A>G	Missense	Hh	Not reported	Not reported	Not reported	Tendon retractions, kyphoscoliosis
[37] (Peng et al., 2019)	*COL6A3*	c.6229G>C, c.5169_5177del	Missense, Deletion	Hh	Not reported	Not reported	Not reported	Mild muscle weakness
[38] (Marakhonov et al., 2018)	*COL6A3*	p.Glu2402Ter	Nonsense	HH	Not reported	Not reported	Not reported	Diffuse muscle weakness, striking distal joint hyperlaxity, proximal contractures, calcaneal protrusion, kyphosis, hip dislocation.
[39] (Collins et al., 2012)	*COL6A3, COL6A2*	c.G6517T, c.G1861A	Missense	Hh	Not reported	Not reported	Not reported	Distal hyperlaxity (finger and ankles), proximal contractures (hips and knees), prominent calcaneus.
[40] (Witting et al., 2018)	*COL12A1*	c.8100 + 2T>C	Missense	Hh	Not reported	Not reported	Not reported	Congenital hip dysplasia
[41] (Punetha et al., 2017)	*COL12A1*	c.8329G>C	Missense	Hh	Not reported	Not reported	Not reported	Upper and lower limbs contractures followed by resolution of contractures, limited motor performance.

HH: homozygous; Hh: heterozygous.

## Data Availability

All the collected data and medical reports used in this study are maintained by the corresponding author and can be provided upon reasonable request.

## References

[1] Huml R.A. (2015). Muscular Dystrophy: Historical Background and Types. Muscular Dystrophy.

[2] Gualandi F., Urciuolo A., Martoni E., Sabatelli P., Squarzoni S., Bovolenta M., Messina S., Mercuri E., Franchella A., Ferlini A. (2009). Autosomal Recessive Bethlem Myopathy. Neuromuscul. Disord..

[3] Bönnemann C.G. (2011). The Collagen VI-Related Myopathies. Handb. Clin. Neurol..

[4] Norwood F.L.M., Harling C., Chinnery P.F., Eagle M., Bushby K., Straub V. (2009). Prevalence of Genetic Muscle Disease in Northern England: In-Depth Analysis of a Muscle Clinic Population. Brain.

[5] Saroja A.O., Naik K.R., Nalini A., Gayathri N. (2013). Bethlem Myopathy: An Autosomal Dominant Myopathy with Flexion Contractures, Keloids, and Follicular Hyperkeratosis. Ann. Indian Acad. Neurol..

[6] Elmas M., Gogus B. (2018). A Case of Bethlem Myopathy with Autosomal Recessive Inheritance with a Novel Mutation in the COL6A2 Gene. J. Biochem. Clin. Genet..

[7] Shih J.A., Folch A., Wong B.L. (2020). Duchenne Muscular Dystrophy: The Heart of the Matter. Curr. Heart Fail. Rep..

[8] Galli F., Bragg L., Meggiolaro L., Rossi M., Caffarini M., Naz N., Santoleri S., Cossu G. (2018). Gene and Cell Therapy for Muscular Dystrophies: Are We Getting There?. Hum. Gene Ther..

[9] Alkuraya F.S. (2014). Genetics and Genomic Medicine in Saudi Arabia. Mol. Genet. Genom. Med..

[10] Bamaga A.K., Alghamdi F., Alshaikh N., Altwaijri W., Bashiri F.A., Hundallah K., Abukhaled M., Muthaffar O.Y., Al-Mehmadi S., Jamaly T.A. (2021). Consensus Statement on the Management of Duchenne Muscular Dystrophy in Saudi Arabia During the Coronavirus Disease 2019 Pandemic. Front. Pediatr..

[11] Alghamdi F., Al-Tawari A., Alrohaif H., Alshuaibi W., Mansour H., Aartsma-Rus A., Mégarbané A. (2021). Case Report: The Genetic Diagnosis of Duchenne Muscular Dystrophy in the Middle East. Front. Pediatr..

[12] O’Brien M. (2023). Aids to the Examination of the Peripheral Nervous System: 6th Edition. Pract. Neurol..

[13] Gowers W.R. (1879). Clinical Lecture ON PSEUDO-HYPERTROPHIC MUSCULAR PARALYSIS. Lancet.

[14] Richards S., Aziz N., Bale S., Bick D., Das S., Gastier-Foster J., Grody W.W., Hegde M., Lyon E., Spector E. (2015). Standards and Guidelines for the Interpretation of Sequence Variants: A Joint Consensus Recommendation of the American College of Medical Genetics and Genomics and the Association for Molecular Pathology. Genet. Med..

[15] Jaganathan K., Kyriazopoulou Panagiotopoulou S., McRae J.F., Darbandi S.F., Knowles D., Li Y.I., Kosmicki J.A., Arbelaez J., Cui W., Schwartz G.B. (2019). Predicting Splicing from Primary Sequence with Deep Learning. Cell.

[16] Jian X., Boerwinkle E., Liu X. (2014). In Silico Prediction of Splice-Altering Single Nucleotide Variants in the Human Genome. Nucleic Acids Res..

[17] Yeo G., Burge C.B. (2004). Maximum Entropy Modeling of Short Sequence Motifs with Applications to RNA Splicing Signals. J. Comput. Biol..

[18] Shihab H.A., Rogers M.F., Gough J., Mort M., Cooper D.N., Day I.N.M., Gaunt T.R., Campbell C. (2015). An Integrative Approach to Predicting the Functional Effects of Non-Coding and Coding Sequence Variation. Bioinformatics.

[19] Kopanos C., Tsiolkas V., Kouris A., Chapple C.E., Albarca Aguilera M., Meyer R., Massouras A. (2019). VarSome: The Human Genomic Variant Search Engine. Bioinformatics.

[20] National Center for Biotechnology Information ClinVar; [VCV000974882.4]. https://www.ncbi.nlm.nih.gov/clinvar/variation/VCV000974882.4.

[21] Lampe A.K., Dunn D.M., Von Niederhausern A.C., Hamil C., Aoyagi A., Laval S.H., Marie S.K., Chu M.L., Swoboda K., Muntoni F. (2005). Automated Genomic Sequence Analysis of the Three Collagen VI Genes: Applications to Ullrich Congenital Muscular Dystrophy and Bethlem Myopathy. J. Med. Genet..

[22] Rs112645828 (SNP)—Genes and Regulation—Homo_Sapiens—Ensembl Genome Browser 111. http://asia.ensembl.org/Homo_sapiens/Variation/Mappings?db=core;r=21:46124962-46125962;v=rs112645828;vdb=variation;vf=1079240881#ENST00000300527_1079240881_G_tablePanel.

[23] Ward A.J., Cooper T.A. (2010). The Pathobiology of Splicing. J. Pathol..

[24] Ward L.D., Kellis M. (2012). Interpreting Noncoding Genetic Variation in Complex Traits and Human Disease. Nat. Biotechnol..

[25] Bateman A., Martin M.J., Orchard S., Magrane M., Ahmad S., Alpi E., Bowler-Barnett E.H., Britto R., Bye-A-Jee H., Cukura A. (2023). UniProt: The Universal Protein Knowledgebase in 2023. Nucleic Acids Res..

[26] Duvaud S., Gabella C., Lisacek F., Stockinger H., Ioannidis V., Durinx C. (2021). Expasy, the Swiss Bioinformatics Resource Portal, as Designed by Its Users. Nucleic Acids Res..

[27] Zhang R.Z., Zou Y., Pan T.C., Markova D., Fertala A., Hu Y., Squarzoni S., Reed U.C., Marie S.K.N., Bönnemann C.G. (2010). Recessive COL6A2 C-Globular Missense Mutations in Ullrich Congenital Muscular Dystrophy: Role of the C2a Splice Variant. J. Biol. Chem..

[28] Full Data View for Gene COL6A2—Global Variome Shared LOVD. https://databases.lovd.nl/shared/view/COL6A2?search_VariantOnGenome%2FDBID=%3D%22COL6A2_000014%22.

[29] 21-46125462-C-G|GnomAD v4.1.0|GnomAD. https://gnomad.broadinstitute.org/variant/21-46125462-C-G?dataset=gnomad_r4.

[30] Wilpert N.-M., Schuelke M., Lala B., Weiß C. (2024). A Mild But Typical Presentation of Bethlem Myopathy With a Novel In-Frame Deletion in COL6A1: Almost Overlooked. Neurology.

[31] Bardakov S.N., Deev R.V., Magomedova R.M., Umakhanova Z.R., Allamand V., Gartioux C., Zulfugarov K.Z., Akhmedova P.G., Tsargush V.A., Titova A.A. (2021). Intrafamilial Phenotypic Variability of Collagen VI-Related Myopathy Due to a New Mutation in the COL6A1 Gene. J. Neuromuscul. Dis..

[32] Kachuei M., Orangi K., Mohammadi A., Mohammadi M., Mojbafan M. (2024). Bethlem Myopathy: A Novel Homozygous Variant of c.385C>T (p.Arg129Cys) in the COL6A2 Gene. Clin. Case Rep..

[33] Baker N.L., Mörgelin M., Pace R.A., Peat R.A., Adams N.E., Gardner R.J.M.K., Rowland L.P., Miller G., De Jonghe P., Ceulemans B. (2007). Molecular Consequences of Dominant Bethlem Myopathy Collagen VI Mutations. Ann. Neurol..

[34] Kutluk M.G., Kadem N., Bektas O., Randa N.C., Tuncer G.O., Albayrak P., Eminoglu T., Teber S.T. (2021). Genotype-Phenotype Correlation of the Childhood-Onset Bethlem Myopathy in the Mediterranean Region of Turkey. Ann. Indian Acad. Neurol..

[35] Oros M., Baranga L., Glangher A., Adina-Diana M., Jugulete G., Pavelescu C., Mihaltan F., Plaiasu V., Gheorghe D.C. (2023). A Diagnostic Challenge in an Adolescent with Collagen VI-Related Myopathy and Emotional Disorder-Case Report. J. Pers. Med..

[36] Stavusis J., Micule I., Wright N.T., Straub V., Topf A., Panadés-de Oliveira L., Domínguez-González C., Inashkina I., Kidere D., Chrestian N. (2020). Collagen VI-Related Limb-Girdle Syndrome Caused by Frequent Mutation in COL6A3 Gene with Conflicting Reports of Pathogenicity. Neuromuscul. Disord..

[37] Peng X.Y., Qu Y.J., Song F., Sun X.F., Ge X.S., Jiao H. (2019). [Clinical manifestations and genetics analysis of collagen type Ⅵ-related myopathy caused by variants in COL6A3 gene]. Zhonghua Er Ke Za Zhi.

[38] Marakhonov A.V., Tabakov V.Y., Zernov N.V., Dadali E.L., Sharkova I.V., Skoblov M.Y. (2018). Two Novel COL6A3 Mutations Disrupt Extracellular Matrix Formation and Lead to Myopathy from Ullrich Congenital Muscular Dystrophy and Bethlem Myopathy Spectrum. Gene.

[39] Collins J., Foley A.R., Straub V., Bönnemann C.G. (2012). Spontaneous Keloid Formation in Patients with Bethlem Myopathy. Neurology.

[40] Witting N., Krag T., Werlauff U., Duno M., Oestergaard S.T., Dahlqvist J.R., Vissing J. (2018). Collagen XII Myopathy with Rectus Femoris Atrophy and Collagen XII Retention in Fibroblasts. Muscle Nerve.

[41] Punetha J., Kesari A., Hoffman E.P., Gos M., Kamińska A., Kostera-Pruszczyk A., Hausmanowa-Petrusewicz I., Hu Y., Zou Y., Bönnemann C.G. (2017). Novel Col12A1 Variant Expands the Clinical Picture of Congenital Myopathies with Extracellular Matrix Defects. Muscle Nerve.

[42] Panadés-de Oliveira L., Rodríguez-López C., Cantero Montenegro D., Marcos Toledano M.d.M., Fernández-Marmiesse A., Esteban Pérez J., Hernández Lain A., Domínguez-González C. (2019). Bethlem Myopathy: A Series of 16 Patients and Description of Seven New Associated Mutations. J. Neurol..

[43] Martins A.I., Marques C., Pinto-Basto J., Negrão L. (2017). Bethlem Myopathy in a Portuguese Patient—Case Report. Acta Myol..

[44] Pino M.G., Rich K.A., Kolb S.J. (2021). Update on Biomarkers in Spinal Muscular Atrophy. Biomark Insights.

[45] El Mouzan M.I., Al Salloum A.A., Al Herbish A.S., Qurachi M.M., Al Omar A.A. (2008). Consanguinity and Major Genetic Disorders in Saudi Children: A Community-Based Cross-Sectional Study. Ann. Saudi Med..

[46] El-Mouzan M.I., Al-Salloum A.A., Al-Herbish A.S., Qurachi M.M., Al-Omar A.A. (2007). Regional Variations in the Prevalence of Consanguinity in Saudi Arabia. Saudi Med. J..

[47] Alkuraya F.S. (2012). Discovery of Rare Homozygous Mutations from Studies of Consanguineous Pedigrees. Curr. Protoc. Hum. Genet..

[48] Metzker M.L. (2010). Sequencing Technologies—The next Generation. Nat. Rev. Genet..

[49] Merlini L., Morandi L., Granata C., Ballestrazzi A. (1994). Bethlem Myopathy: Early-Onset Benign Autosomal Dominant Myopathy with Contractures. Description of Two New Families. Neuromuscul. Disord..

